# Some Questions as to Rank in the Army Medical Service

**Published:** 1917-08

**Authors:** 


### Some Questions as to Rank in the Army Medical Service.

There is a wide spread misconception that the Medical Department of the U. S. Army consists of a small body of men not imtimately connected with military administration, not executives except in the sense that any organization involves subdivision of authority to conduct its own affairs and in that applying to the professional ministrations of any physician, more especially not holding commands in the military sense, indeed not even having the men over whom they might hold authority, and not combatants. With this notion in mind, the commissioning of medical men in the army and their designation by military titles are regarded, respectively, as a convenient compromise for the sake of allowing the payment of salaries necessary to secure the services of professional men, and as a sort of unmerited and purely honorary degree.

Conceding, at the outset, the considerable and inevitable differences between line and staff service, an examination of facts may prove enlightening. Beside the three general branches of infantry, cavalry and artillery, there are 13 special departments of the army of small magnitude and for which there is no general term in common use. Seven of these number well within a hundred and of these, several consist practically entirely of officers detailed for special duties, with such clerical help as is necessary and which is, for the most part, not included as part of the army personnel. The Military Academy, inclusive of 706 cadets numbers about 1300; (statistics of 1916); the Ordnance Dept. 850; the Signal Corps 1578; the Engineers 2,190; the Medical Dept. 4,616; the Quartermaster's Dept. 6,590. It will thus be seen that the Medical Dept. ranks second in numbers among what many persons regard as army accessories. It is almost certain that it will be increased many times in numeric strength within the next few months, and its increase in commissioned officers will be relatively greater than that of any other branch of the army, on the basis of ultimate numeric strength; and it is not at all improbable that it will become the largest branch of the army aside from the line.

One of Kipling's characters speaks scornfully of a staff officer as "The Colonel Sahib without a regiment." Many persons have believed that this thought applied especially to the medical officer but, in general, it applies less strictly to this department than to other staff organizations. In 1916, the whole number of commissioned officers was about 1/21 of the total army; the number of medical officers about 1/8 of the total medical force. With due allowance for men coming under the authority of medical officers in a purely professional way, there is no question but that the medical officer commands as many men as the average line officer. If we consider the authority of general officers over commissioned officers of lower rank, which is a rather fairer method of weighing authority and responsibility, we find that the Surgeon General (Major General for the last two years, previously Brigadier General) has an extent of command at least 50% greater than that of the average. It is, of course, impossible to foretell the future but it is highly probable that, within the next year, the Surgeon General will exercise a command over men, equal to that of a Major General commanding a division and over officers, equal to that of the commander of a field army.

Near the other extreme, consider the command of the Captain of an ambulance company: 4 1st lieutenants, 1 1st sergeant, 11 sergeants, 5 mechanics, 2 cooks, 2 assistant cooks, 20 chauffeurs, 2 musicians, 43 privates; total 90. On a numeric basis, his command is about 2/3 that of a captain of the line on the nominal strength of a company; but it is far greater in numbers if we allow for the wounded coming under his care; greater in responsibility if we regard merely the rank and distribution of the men under him; enormously greater either from the standpoint of property responsibility or of personal initiative when we consider the relatively greater degree of independence of next higher officers.

It is true that, outside of his own department, the medical officer does not exercise command, even under circumstances in which a line or staff officer would supersede another or assume charge of troops without a leader. For example, we know a captain of Volunteers during the Civil War who claimed to have commanded a division for a brief time. In many respects, however, a medical officer exercises the general influence of any other and it is reported that, in emergencies, they have led, though they could not formally assume command of bodies of troops.

The medical officer is not a combatant in the strict sense of the word but, under modern conditions, he incurs the hazards of war to a degree in some respects greater than that of others, especially if we exclude subordinate officers of the line. For example, the experience of the British Army in the present war is that officers have a greater mortality than privates and that, of all officers, the medical have the highest mortality and, strangely enough, those of the aviation corps, the lowest. No officer is, under modern conditions, expected to be a direct factor in combat and if a distinction is to be made at all with regard to combatancy, it would perhaps be less unjust to draw it according to the safeguards properly attending high rank or to apply it to special branches of the service whose duties afford greater exemptions from danger than those of the medical department. The fact is that any such distinction is essentially unjust, wherever and whatever the arbitrary line drawn.

There is one phase of the matter of military rank that may not have occurred to line officers who are reluctant to recognize it in members of the medical department. The medical man has had a degree and extent of educational and technical training fairly comparable with that of a West Point graduate though undoubtedly less onerous during its reception; still more closely comparable with that of a professional engineer or other military specialist. More than this, the selection of applicants has, for many years, taken only a small minority of candidates for appointment as assistant surgeon and the knowledge of this fact has deterred the vast majority of medical graduates from even applying for examination. The army doctor has, deep down in his heart, the pride of the honor man among the line and other departmental officers. At the present time, a great many of the volunteers for army medical service, aside from the very youngest, are men who are, from patriotic motives, or at the worst, motives connected with efforts at technical improvement or desire for a change, accepting a rank considerably below what they would now hold if they had entered the army as a career and, very likely, still further below the rank which corresponds, in pay and prestige to what the title "Doctor" now means to them. Such men are not at all apt to insist on the use of a military title as a matter of bumtiousness, though they may do so in the natural attempt of all beginners to conform to the letter of requirements.

The rank of the medical corps, with reference to that of other branches of the service, seems to have been quite well adjusted in this country. The lowest grade is one higher and the highest one lower than for the army as a whole and the proportion of officers of different rank corresponds fairly well with the general figures. In the rapid expansion now in progress, special problems arise. For example, men of practically no military experience are undergoing training with the expectation of receiving commissions of the three lower grades, immediately, somewhat according to age. If for example a young man, with a high school education and no special training, is to be commissioned a captain in the line or of engineers, it does not seem fair to commission an older man, with a college education and a technical training in medicine, a first lieutenant. On the other hand, it would be equally unfair to the regular medical officer to place a volunteer from civil practice in the same rank that he himself has won by years of hard work and attention to duty. There does not seem to be, however, the degree of candid political favoritism in the appointments, that was manifested in the Spanish War and the credit for this is largely due to the foresight of the Medical Department in inaugurating the system of a Reserve Corps, which has recently been extended to the army as a whole. None of these problems can be solved satisfactorily to all and without delay. One of the tests of patriotism is to make practical allowance for this fact.

(Note. This article has been submitted to the Surgeon General's Office for criticism and has been recommended for publication, with a minor correction).

